# Inflammatory complications of vocal fold injection with hyaluronic acid: a multiinstitutional study

**DOI:** 10.3906/sag-2008-5

**Published:** 2021-04-30

**Authors:** Necat ENVER, Elad AZİZLİ, Sevtap AKBULUT, Emel ÇADALLI TATAR, Muhammed Kürşat YELKEN, Kayhan ÖZTÜRK, Hakan COŞKUN, Ahmet Hakan BİRKENT, Zahide Çiler BÜYÜKATALAY, Ozan Bağış ÖZGÜRSOY, Haldun OĞUZ

**Affiliations:** 1 Department of Otolaryngology, Pendik Training and Research Hospital, Marmara University, İstanbul Turkey; 2 Department of Otolaryngology, Private Practise, İstanbul Turkey; 3 Department of Otolaryngology, Faculty of Medicine, Yeditepe University, İstanbul Turkey; 4 Department of Otolaryngology, Dışkapı Yıldırım Beyazıt Research and Training Hospital, University of Health Sciences, Ankara Turkey; 5 Department of Otolaryngology, Faculty of Medicine, Maltepe University, İstanbul Turkey; 6 Department of Otorhinolaryngology, Medicana Konya Hospital, Faculty of Medicine, KTO Karatay University, Konya Turkey; 7 Department of Otolaryngology, Faculty of Medicine, Bursa Uludağ University Turkey; 8 Department of Otorhinolaryngology, Nişantaşı University, İstanbul Turkey; 9 Department of Otorhinolaryngology Head and Neck Surgery, Faculty of Medicine, Ankara University Ankara Turkey; 10 Department of Otolaryngology, Fonomer, Ankara Turkey

**Keywords:** Vocal fold injection, hyaluronic acid, hyaluronic acid with dextranomer, inflammatory reaction, vocal fold paralysis, sulcus vocalis

## Abstract

**Background/aim:**

This study aimed to assess the inflammatory adverse reactions of vocal fold injection laryngoplasty with hyaluronic acid.

**Materials and methods:**

This study was a retrospective chart review of patients who underwent vocal fold injection augmentation with HA injection from January 2005 to September 2016 in nine different institutions. Demographic data, indication for injection, injection techniques, types of injection material, settings of procedure, and complications were reviewed. The types of complication, onset time, and management of complications were also noted.

**Results:**

In all, 467 patients were identified. The majority of patients had been injected under general anesthesia (n = 382, 84.7%). For injection material, two different types of hyaluronic acid were used: hyaluronic acid alone or hyaluronic acid with dextranomer. Complications occurred in nine patients (1.9%). The majority of complications were inflammatory reactions (n = 7, 1.47%). Main symptoms were dysphonia and/or dyspnea with an onset of 0 h to 3 weeks after the hyaluronic acid injection. Three patients were hospitalized, one of which was also intubated and observed in the intensive care unit for 24 h. Systemic steroids and antibiotics were the main medical treatment in the majority of cases. There was no statistical difference in complication rates between patients who received hyaluronic acid and those who received hyaluronic acid with dextranomer (P = 0.220).

**Conclusion:**

Hyaluronic acid can be considered as a safe substance for the injection of vocal folds with a low risk of inflammatory reaction.

## 1. Introduction

Glottic insufﬁciency is one of the most common etiologic factors of dysphonia. Glottic insufﬁciency is usually secondary to unilateral vocal fold paralysis, unilateral or bilateral vocal fold paresis, sulcus vocalis, and presbylaryngitis. Injection laryngoplasty is a common therapeutic option for treatment of glottic insufficiency. The purpose of injection is to gain adequate glottic closure to alleviate phonatory and swallowing symptoms [1]. Although several injectable substances have been in use for the larynx since the inception of injection laryngoplasty in 1911, the ideal one is yet to be found [2]. 

Hyaluronic acid (HA) is among the most commonly used substances for injection laryngoplasty. Its ease of injection and unique properties in tissue regeneration, namely recruitment of fibroblasts, deposition of collagen, and improvement of the viscoelastic properties of the injected tissues, have made it a popular injectable material [3]. This naturally existing polysaccharide is found in the extracellular matrix of human cells and is also abundant in the vocal fold lamina propria. It is biocompatible and rarely induces foreign body reactions or cell-mediated immune responses. Clinical studies have supported the safety and efficacy of HA for vocal fold augmentation [4]. 

HA has been demonstrated as a safe material for vocal fold injection in the literature; however, most of these studies have small sample sizes. There has been case reports about inflammatory adverse reactions after vocal fold HA injections [5–7]. Recently, a study with a large sample size published the results from the assessment of 186 patients from a single institution [8]. In our study we aim to gather the clinical data of several institutions to understand the presentation and management of inflammatory adverse reactions of HA. The goal of our study is to identify the rate of complications and the adverse reactions after HA injection laryngoplasty in a multiinstitutional setting.

## 2. Materials and methods

A retrospective chart review of all patients from nine institutions who underwent vocal fold injection laryngoplasty with HA alone (Restylane, Galderma/Q‐Med, Uppsala, Sweden) or HA with dextranomer (HA-D; Dexell, Istem Medikal, Ankara, Turkey) from January 2005 to September 2016 was performed. Injections were done either unilaterally or bilaterally in one session. The side and volume of injectable materials were decided according to clinical decision of the physician. A retrospective chart review was performed to identify patients with local complications. Patients with previous laryngeal surgery, vocal fold injections and patients with radiated neck were excluded from the study.

Demographic data, injection technique, type of injection material, the indication for injection, location of the procedure, and occurrence of complications were reviewed. Type, onset, and management of complications were also noted for patients with complications. Results were grouped according to the location of the procedure: under local anesthesia in the office setting (office group) or under general anesthesia in the operating room via direct microlaryngoscopy (OR group).

Complication rates were compared according to injection materials and the technique of injection. Statistical analysis of the study was performed using the MedCalc Statistical Software version 12.7.7 (MedCalc Software bvba, Ostend, Belgium). The differences between groups were compared using the chi-squared test (or Fisher’s exact test when applicable).

## 3. Results

A total of 476 patients underwent laryngeal HA injection over an 11-year period in nine institutions. The average age of patients was 47.1 ± 13.7 years (range = 19–70), and the only indications were vocal fold paralysis (n = 417) and sulcus vocalis (n = 59). In all, 403 patients (84.6%) were injected with HA-D, and 73 (15.4%) were injected with HA only. The majority of injections were done under general anesthesia (OR group) (n = 382, 84.7%).

Complications were seen in nine patients. The mean age of patients with complications was 46.7 years (range = 36–57), and six of these patients were women. Five of the patients who experienced complications were in the OR group and received the injection under general anesthesia, whereas the remaining four were in the office group. There was no significant difference between complication rates in the office group and those in the OR group (P = 0.08). The main indication was unilateral vocal fold paralysis (n = 7), whereas two of the patients had sulcus vocalis. Although all the patients with complications were injected with HA-D, there was no statistically significant difference between patients who received HA and those who received HA-D (P = 0.220).

**Figure 1:**
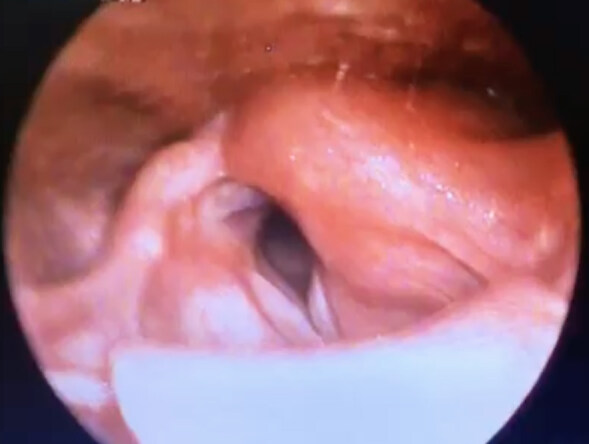
Flexible laryngoscopy showing edema at the injection site, left arytenoid (star), and aryepiglottic fold (arrow) of patient 1, one day after injection.

**Figure 2:**
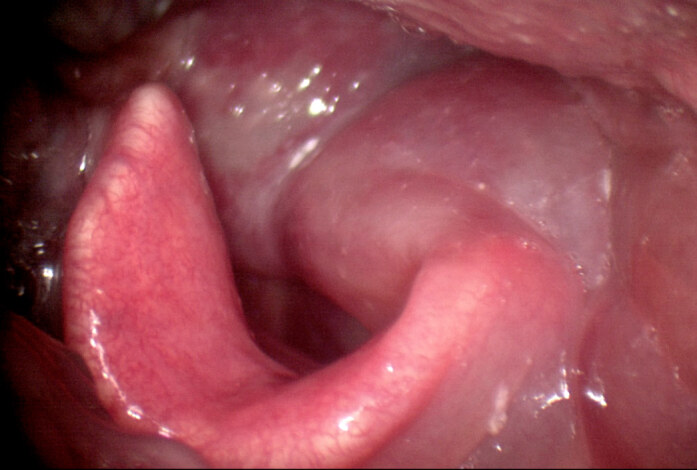
Laryngoscopic examination of patient 8 one day after injection, demonstrating severe left vocal fold (star), left aryepiglottic fold (black arrow), and left arytenoid edema (white arrow).

**Figure 3:**
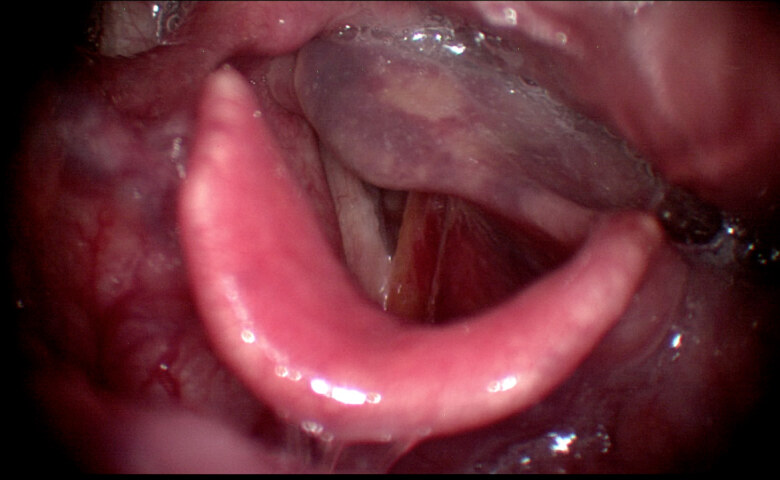
Laryngoscopic examination of patient 8 three days after injection with decreased edema in the larynx after 2-day hospitalization.

Five patients with complications presented mainly dyspnea, and the other four presented the chief complaint of dysphonia. The symptoms were observed postoperatively within the first 24 h in the majority of patients (n = 5). However, it was observed postoperatively on the second day in two patients, and on the third week in one patient. In videolaryngostroboscopic examination, the most common findings included hyperemia and edema of the vocal folds with or without false vocal folds, which were observed in seven patients, although the severity of these inflammatory findings varied (n = 7). Arytenoids were inflamed in five of these seven patients, and movements of the vocal folds were impaired in three of them, although their vocal folds were mobile preoperatively (Figures 1–3). In one patient, VLS examination revealed vocal fold hematoma that decreased mucosal wave and amplitude in one side of the larynx. In another patient, superficial deposition of the injected material on the vocal fold were noted, which also caused decreased mucosal wave and irregular closure in the affected vocal fold (Table).

**Table:** Clinic data of the patients with complications.

Case	Age	Sex	Approach	Diagnosis	Symptom	VLS Exam	Onset	Treatment	Interventions	Resolution time
1	50	M	Transoral (GA)	UVFP	Dysphonia	Vocal fold edema(Figure 1)	24 h	Clinic follow-up	-	2 weeks
2	44	F	Transoral (GA)	UVFP	Dysphonia	Vocal fold edema	24 h	Antibioticsteroid(inhaler)	-	60 days
3	42	M	Transoral (GA)	Sulcus vocalis	Dysphonia	Superior transposition of material	0 h	Clinic follow-up	90th day surgery	90 days
4	54	F	Transoral (GA)	UVFP	Dysphonia	Inflammation edema in FVF, arytenoid, and VF	24 h	Antibioticsteroid	Intubation, ICU (1 day)Hospitalization (totally 2 day)	3 days
5	46	F	Thyrohyoid (LA)	Sulcus vocalis	Dyspnea	Inflammation and edema in FVF, arytenoids, decreased VF motion	3 weeks	Systemic steroid	2 ED visit1-night hospitalization	3 weeks
6	57	M	Transoral (GA)	UVFP	Dyspnea	Bilateral inflammation edema in FVFs, arytenoids, decreased VF motion	0 h	Systemic steroid	2 days hospitalization(1-night ICU)	4 days
7	36	F	Thyrohyoid (LA)	UVFP	Dyspnea	Inflammation edema in FVF, arytenoid	72 h	Oral steroid and antibiotic	-	1 week
8	55	F	Transoral (LA)	UVFP	Dyspnea	Inflammation edema in FVF, arytenoid (Figures 2 and 3)	24 h	Systemic steroid	2 days hospitalization	2 day
9	37	F	Cricothyroid (LA)	UVFP	Dyspnea	Hematoma	0h	Clinic follow-up	-	2 days

Six of the patients with local inflammatory reaction received the treatments. Oral or parenteral corticosteroid was used as the main treatment in every patient who received treatment, and three of these patients received additional antibiotic treatment. Four of the patients needed 1 or 2 days of hospitalization. Two of them were kept under observation in the intensive care unit for 24 h; of these two patients, one underwent orotracheal intubation. All symptoms resolved between 2 days to 3 weeks in these patients.

Patients who demonstrated no signs of inflammation received no acute treatment or hospitalization. The patient with vocal fold hematoma was observed without treatment, and the hematoma resolved spontaneously in 2 weeks, completely preserving the integrity of mucosal wave and amplitude. However, the patient with superficial deposition of the injected material underwent microlaryngoscopic surgery for removal of the deposit 3 months later.

In our patient series of 476 individuals, the overall complication rate was 1.9%, (9/476), and the inflammatory complication rate was 1.47% (7/476). There was no difference in terms of inflammatory complication rates between office (n = 3) and OR (n = 4) groups (P = 0.142). Although all the inflammatory complications were only seen in HA-D injected patients, there was no statistically significant difference for these complications between patients treated with HA and those treated with HA-D (P = 0.309)

## 4. Discussion

HA is a commonly used material in injection laryngoplasty. However, it only lasts approximately 3 to 6 months [4]. In our patient series, two types of injection material were used: HA and HA-D. HA with dextranomer aims to increase the duration of the material with the permanence of the positive load of dextranomer. This form of HA is frequently used in urology for vesicoureteral reflux treatment [9]. No known inflammatory side effect with HA-D in urological practice has been documented in the recent literature [9]. Oguz et al. demonstrated the safety and efficacy of HA-D for laryngeal injections in a small group of patients [10]. Similarly, in our series, there was no significant difference for complication rates between HA and HA-D.

Injection laryngoplasty has been a workhorse in laryngology since it was ﬁrst described by Brunings [2]. During the last century, with the improvements in general anesthetics, surgeons now prefer the operating room for injections. However, in the last 20 years, there has been a rising trend of in-office laryngeal injections [11]. The safety of the office-based injection is widely accepted in the literature [5,11]. In our study, only a small group of patients were injected in the office (n = 69, 14.5%), and four out of nine complications (44.4%) were experienced by patients who received office-based injections. There was no significant difference for overall complication rates between office-based injections and operating room injections in our series. Furthermore, for inflammatory complications, there was also no difference between these groups. Complication rates for injection laryngoplasty are very low, and to claim definitive conclusions, larger multiinstitutional studies are needed [5].

Complications that may be associated with vocal fold HA injection can be divided into two main groups: technical problems and inflammatory problems. Technical problems are related to the applied volume, application depth, and application area. Of the nine patients included in our series, two of them (22%; cases 3 and 9) experienced complications that could be considered as technical problems, such as hematoma and submucosal injection. Cases involving such technical problems have been described in the literature [7,8].

Besides the technical problems mentioned above, seven out of nine patients in our series experienced complications that can be classified as inflammatory. In animal studies, mild inflammation has been observed after vocal fold HA injection; however, no necrosis or granuloma formation has been observed [12]. In the literature, a few case series and case reports have indicated inflammatory adverse reactions following vocal fold HA injection [5–7]. In the largest series published by Dominguez et al., the incidence of inflammatory complications was 3.8%. In our patient group, this rate was 1.47% [8].

The onset of inflammatory reaction after vocal fold HA injection varies in the literature. Although in some of the series, there were patients whose symptoms started right after or several hours after injections [5,8], the most common onset time was 2 or 3 days after injection laryngoplasty in most of the series [7,8]. Very rarely, the start of the symptoms could be delayed for up to 3 weeks. Our results were similar with those recorded in the literature.

Various side effects associated with dermal HA injection have been described in the literature. These side effects include erythema, edema, and irritation to foreign body granuloma formation, ulceration, necrosis, and hypersensitivity reactions. The frequency of these side effects ranges between 0.06% and 0.8% in dermal applications [13,14]. HA may cause adverse inflammatory reactions through three main mechanisms: an ischemic event, an allergic or hypersensitivity reaction, or an acute infection of bacterial origin that causes inflammation with fluctuant and erythematous nodules [15].

Vasoconstriction may be one of the possible reasons for injections in the larynx because of a region limitation by cartilage from the lateral. In the case of overinjection, especially after radiotherapy, compression may occur in the vascular structures of the larynx, which may lead to ischemia. HA injections that cause vascular compression in dermal injections usually show an acute whitening followed by regional necrosis and ulceration in postoperative hours [13]. There is one report in the literature of a suspected compartment syndrome of the hemilarynx after injection with HA in a patient with a history of radiation [16]. Although patient with a history of neck radiotherapy was excluded in our study, there are no complications in this patient subgroup in Dominguez et al.’s series [8]. The injected volume of the material could be a factor leading possible cause of vascular compression. Unfortunately, there is data about the volume of the augmentation in our series. Even though in Dominguez study, the volume of injected materials in patients with inflammatory complication is within the range of average amount of injected HA, the relation between the amount of the injected HA and the inflammatory complication is not clearly understood yet. 

Hypersensitivity due to bacterial proteins can be observed in relation to the production technique. HA-based injectable material contains very small amounts of protein, which can cause some reactivity. Currently, purification of HA fillers is considered to be more effective than before. However, the alleged reasons for hypersensitivity are less consistent. HA fillers might still contain trace amounts of protein contaminants even after purification. Therefore, hypersensitivity is still the most important pathophysiological cause [17]. 

In our patient series, HA-D was used for all patients with inflammatory complications. Inflammatory complication rates in our study were 1.9%. Although no statistical difference was demonstrated between HA-D and HA injections, it can be speculated that dextranomer may be the trigger for hypersensitivity. Even though no inflammatory side effects have been shown in the literature from urological practice, because of the location of the injection site, the possibility may be easily underestimated. In the published clinical series by Dominquez et al., none of the augmentations include dextranomer or any other cationic substance. The inflammatory complication rate of their study (3.8%) [8]. was also similar to ours. Another case series of vocal fold HA injection demonstrated an inflammatory complication rate of 4.7% over 62 injections [18].

Infection is an important complication that must be avoided in dermal HA injections. This kind of complication is rare in dermal applications [16]. In patients with infection, increased white-blood-cell (WBC) and C-reactive protein (CRP), and/or abscess formation would be expected. Treatment with antibiotics and hyperbaric oxygen therapy after drainage has been demonstrated as an effective treatment approach. In cultures after abscess drainage, staphylococcal and streptococcal species were detected most frequently in facial application of HA [14]. 

Although none of the patients with complications exhibited WBC count elevation, empiric parenteral antibiotic therapy was initiated in all individuals with inflammatory findings in our patient group. This occurred because a significant proportion of patients applied with a disturbing complaint, such as dyspnea, and all possible treatment options were provided empirically. 

In the literature, only one case of laryngeal abscess after vocal fold HA injection has been reported. In that case, the patient had a high WBC count and constitutional symptoms, and no organism was revealed in the microbiological culture and gram stain from the abscess sample. The patient’s symptoms were relieved after drainage, systemic antibiotic, and corticosteroid therapy [15]. 

The most commonly used treatment among our patients was the corticosteroid. If it is accepted that inflammatory complications happen as a result of hypersensitivity reactions or infections, which are the most likely pathophysiologic explanations, we can speculate that corticosteroids may have been helpful in decreasing edema and inflammation in our patients. Although none of the patients in our study had higher WBC or C-reactive protein, some of them also received empiric antibiotic treatment. In the facial application of HA, when a patient has an inflammatory reaction, antibiotics are widely used with hyaluronidase and corticosteroids [14]. Hyaluronidase would be beneficial to decrease bulkiness in overinjection complications; however, while working on the glottic level, it would cause an acute reduction in the size of the airway. 

Surgery is also an option for patients with severe dyspnea resulting from HA injection. Tracheotomy may be needed in an advanced airway obstruction. In our series, only one patient with bilateral injection was intubated and hospitalized in intensive care for 1 day, but none of our patients had a tracheotomy performed. Only one patient (case 3), who had transposition of injection material in the vocal fold, received surgical intervention in our study. 

Although medical treatment and surgery are possible treatment options that can be offered for inflammatory complications of vocal fold injection, none of these treatments are evidence-based. Future studies are needed to clarify special treatment modalities for inflammatory complications of vocal fold injection.

To the best of our knowledge, our study is the largest vocal fold HA injection series ever published. Dominguez et al. published the results of 186 patients from a single institution [8]. Gathering and assessing complications from different institutions increases the possibility of identifying appropriate approaches to these rare complications. 

Multiinstitution retrospective studies have some disadvantages as well. It is not easily possible to standardize the treatment and management protocols. Each clinic has a different method of archiving patients’ data. This can limit the quality of information obtained from retrospective studies such as ours. Patients’ perceptual and acoustic voice analyses could be used in a prospective setting. Absence of patient-centered questionnaires was also another limitation of our study. These indices are widely used in laryngology clinics. They are especially useful to observe problems from patients’ perspective and to compare the results of different institutes.

## 5. Conclusion

Injection laryngoplasty with HA is a common therapeutic option for the treatment of glottic insufficiency. In our series, complications of all types were found to occur at a rate of 1.9%. Dyspnea and dysphonia were the common symptoms of complication most commonly starting after 1 day. Corticosteroid and antibiotic are the most accepted treatment by the authors of the study. HA can be considered a safe substance for vocal fold injections.

## Informed consent

The study protocol was approved by the institutional review board of Marmara University Medical Faculty, where the de-identified data were collected and analyzed, and by all contributing institutions administrations (Marmara University Clinical Researches Ethic Committee Ref No: 09.2019.301). 
